# Psychological adjustment of men with prostate cancer: a review of the literature

**DOI:** 10.1186/1751-0759-1-2

**Published:** 2007-01-10

**Authors:** Sidney Bloch, Anthony Love, Michelle Macvean, Gill Duchesne, Jeremy Couper, David Kissane

**Affiliations:** 1 Department of Psychiatry, University of Melbourne, Melbourne, Australia; 2 Department of Psychology, La Trobe University, Melbourne, Australia; 3 Victorian Cancer Council, Melbourne, Australia; 4 Peter MacCallum Cancer Centre, Melbourne, Australia; 5 Department of Psychiatry and Behavioural Sciences, Memorial Sloan-Kettering Cancer Centre, New York, USA

## Abstract

**Objective:**

Prostate cancer (PCA) is the most common malignancy and a major cause of death in men but, importantly, a substantial proportion will live for several years following diagnosis. However, they face the prospect of experiencing symptoms, side-effects of treatment and diminished quality of life. The patient's psychological adjustment is particularly complex, given the potential trajectory of the disease, from the point of diagnosis, with its immediate impact, to the phase of palliative care, with its attendant issue of facing mortality. Since a comprehensive review of the literature on psychological adjustment of men with PCA has not yet been done, we have documented relevant research, integrated findings and drawn conclusions, where possible, in order to map out clinical and research implications.

**Method:**

We searched 5 databases for the period 1994 – July 2006, during which most of the work in the field has been done.

**Results:**

We found few studies of substance among the 60 we examined to draw conclusions about psychological adjustment to prostate cancer and its treatment. This is in marked contrast to the picture in breast cancer. While some patterns have emerged, many gaps remain to be filled.

**Discussion:**

Aspects of methodology need attention, particularly longitudinal, prospective designs, incorporation of control groups and the use of valid and reliable measures. There is scope for qualitative studies as a complement to quantitative research.

## Background

Prostate cancer (PCA) is the most common malignancy and a major cause of death in men but, importantly, most patients will live for a decade or longer following diagnosis. Consequently, they face the prospect of experiencing symptoms, side-effects of treatment, and diminished quality of life [1]. Research on psychological adjustment to PCA suggests that it is related to factors such as the stage of the cancer, time since diagnosis, and type of treatment given. The latter is especially noteworthy, given the range of interventions, which include watchful waiting, radiation treatment (external beam and brachytherapy), surgery and hormonal therapy.

Puzzlingly, almost all research has been devoted to physical aspects and side-effects of treatment and very little to such psychological features as emotional distress, coping and psychiatric morbidity. Psychological adjustment is complex, given the disease's potential trajectory – from the point of diagnosis, with its immediate impact, to the phase of palliative care, with its attendant existential issues. Since a focused review of the literature on psychological adjustment has not yet been published, we have documented what is known about this important aspect, integrated pertinent findings and drawn conclusions, where possible, in order to map out clinical and future research implications.

In doing so, we extend the contribution of Eton and Lepore [2], who reviewed studies on health-related quality of life, both PCA-specific and general. They mainly paid attention to physical aspects (urinary, sexual and bowel) but psychosocial associations were also addressed. They confined their coverage to three types of studies: cross-sectional, longitudinal and those incorporating a comparison group. We have taken a different approach by concentrating on psychological adjustment specifically but refer when necessary to the physical domain. Kiss and Meryn [3] have compared psychosocial aspects of PCA and breast cancer but cited only a small proportion of the studies published. Roesch et al. [4] have conducted a meta-analytic review of coping with PCA, to which we refer below.

## Method

We searched five databases: Medline, PsycINFO, Biological Abstracts, Sociological Abstracts and Cumulative Index to Nursing and Allied Health (CINAHL), for the period 1994 – July 2006, during which period most of the research had been done. Given the lack of a review on the ways in which men with PCA deal with psychological aspects of the experience, we opted to focus on the most pertinent aspects of psychological and psychiatric morbidity, coping and adjustment, and the potential effects of psychosocial (interventions to assist patients to manage effectively) used combinations of the following terms: prostate cancer, psychological adjustment, psychiatric, psychosocial, morbidity, distress, depression, anxiety, coping, and quality of life (QOL). A manualized search of relevant references cited in the identified articles followed. Given the brief history of systematic research, we opted to be inclusive but only incorporated studies with reasonably sized and unbiased samples and appropriate designs, including adequate descriptions of instruments used. Thus, we have excluded, for instance, case histories and anecdotal reports. We also omitted the small number of non-English articles, research on couple and family aspects (the subject of a separate review in press), and studies that combined two or more cancer groups where it was impossible to comment on the PCA subgroup separately. We examined unpublished dissertations but did not find any of notably original substance; it is conceivable that, if any of this work reaches peer-reviewed publication, it will then warrant attention of future reviewers.

We identified 60 reports, covering a range of topics. In order to evaluate the material systematically, we partitioned it into five coherent categories: cross-sectional, longitudinal, coping, PCA treatments and their psychological effects and the impact of psychological interventions. Given the constraints of space, we have created 8 tables which contain the main elements of the studies we necessarily have had to consider schematically in the text. After addressing each in turn, we have concluded with a critique of the overall endeavour and a discussion of clinical and research implications. We had entertained conducting a meta-analysis of intervention studies but concluded this was unwarranted in that there were very few controlled trials, and almost all of them were relatively unsophisticated conceptually.

### Cross-sectional studies of psychological adjustment to PCA (see Table 1)

**Table 1:** Cross-sectional studies of psychological adjustment to prostate cancer

**Study**	**Design**	**Characteristics of the sample**	**Major Findings**
Bacon et al. (2002)	• Retrospective comparison of patients and aged-matched healthy controls using the SF-36, UCLA Prostate Cancer Index and the CARES-SF	• 783 men with PCA (localized disease), no breakdown by treatment; 1928 age-matched controls	• Patients had poorer sexual, urinary and bowel functioning but not role function or mental health • Patients reported more bother from sexual, urinary and bowel functioning • Symptoms were related to both physical and psychosocial QOL domains
Balderstone and Towell (2003)	• Retrospective study of distress using FACT-P and the Hospital Anxiety & Depression Scale	• 94 men with PCA in various stages	• Prevalence of distress: 38% • Distress was related to poorer physical functioning and lower social support
Clark et al. (2003)	• Retrospective comparison of patients and normal controls using the SF-12 and purpose-made symptom measures	• 349 men with early-stage PCA and 398 controls	• Bowel, urinary and sexual symptoms created greater bother for men with PCA than for controls • Bowel, then sexual, symptoms had greatest impact on QOL
Curran et al. (1997)	• Baseline measures of QOL (EORTC-QLQ) in patients with advanced PCA	• 638 advanced-stage PCA patients in 1 of 3 groups: Locoregional; Poor prognosis metastatic; Hormone resistant	• Four scales distinguished between the 3 groups • Locoregional group had better QOL than metastatic, or hormone resistant group • Some physicians under-rated patients' level of pain
Heim and Oei (1993)	• Retrospective study using the McGill Pain Questionnaire, Beck Depression Inventory, and State-Trait Anxiety Inventory	• 47 patients; 80% described as 'non-metastatic'	• 43% reported pain; 20% reported depression • Pain was correlated with depression and anxiety, increased use of analgesics, and later stages of disease
Helgason et al. (1996)	• Retrospective observational study of PCA patients and age-matched controls' sexual, urinary and bowel functions using the Radiumhemmet Scale of Sexual Function	• 342 patients with mixed stage and treatment status, compared with 319 controls of similar age	• Both groups experienced decline in sexual functioning but more PCA patients were severely distressed • Urinary and bowel symptoms were less common and few were severely distressed as a result of them
McBride et al. (2000)	• Cross-sectional study – mail-out to PCA and breast cancer patients using Impact of Events Scale and measures of lifestyle activities	• 920 (420 PCA; 93% radical prostatectomy. 500 were breast cancer patients) usable responses from 1667 questionnaires distributed	• Breast cancer patients were younger, sicker and had higher trauma scores • Trauma scores were negatively related to time from diagnosis • Among the PCA patients, regular exercisers had lower trauma scores
Schag et al. (1994)	• Retrospective observational study of QOL in cancer survivors using CARES	• 278 survivors (disease free); 57 lung, 117 colon and 104 PCA	• QOL improved for colon cancer but decreased for PCA survivors • All groups reported a range of QOL issues
Stone et al. (2000)	• Retrospective comparison of fatigue and depression in cancer patients and healthy controls using EORTC-QLQ-30 and HADS	• 227 cancer patients, including 62 with PCA; 98 controls	• Fatigue is common in cancer, especially those with advanced disease • Fatigue related to depression, anxiety, pain, dyspnoea

Studies by Bacon et al. [5], Clark et al. [6] and Helgason et al. [7] stand out since the samples are adequate and compared with controls. The Helgason team reported diminished sexual function for both but that a greater proportion of the men with PCA were 'severely distressed' by this. Both Clark's and Bacon's groups found patients more bothered than controls by bowel, urinary and sexual symptoms, but mental health scores were similar in the two groups in Bacon et al. In essence, although affected by symptoms, patients did not report more distress than did healthy men. Clark et al. found that psychological adjustment was as affected as physical status. Questionnaires developed for the latter study revealed greater complexity than had previously been discerned. For example, certain treatments were associated with patients' confidence that the cancer was under control although they still felt a loss of intimacy and masculinity.

Three papers have concentrated on depressive symptoms. Heim and Oei [8] found one in five patients reporting depression, half of these at the 'severe' level. Depression correlated strongly with ratings of pain. The vaguely described sample of convenience (80% labelled 'non-metastatic') is a distinct limitation. Stone et al. [9] observed severe levels of fatigue related to depression, especially in men with advanced disease. Although the study included controls, the sample was small, precluding generalisation of the finding. Balderson and Towell [10] assessed, retrospectively, 94 men at various stages of PCA. Distress, as reflected in mixed depression and anxiety in 38% of them, was associated with poorer social, physical and functional well-being. As this was a sample of patients seeking support, it is difficult to generalise the findings.

Researchers have tended to view PCA patients as a homogeneous group but subgroups, for example men with localised early-stage disease (often asymptomatic) and metastatic disease (usually linked to recurrence), are identifiable. Different psychological reactions can be anticipated. Curran et al. [11], for instance, compared men with localised, metastatic and hormone-resistant cancer. Those with localised disease had better adjustment than the advanced groups.

Patients with long-standing PCA grapple with several psychological challenges. Schag et al. [12] studied this in a convenience sample diagnosed five or more years earlier. The men, particularly those with a co morbid physical or psychiatric disorder, reported a decline in psychological functioning, with escalating distress and cognitive impairment.

McBride et al. [13] examined the psychological effects of a cancer diagnosis using the Impact of Events Scale [14]. Strengths included the large sample of 420 men, most of whom underwent surgery. A briefer period since diagnosis and regular exercise were both related to lower levels of psychological distress. Penedo et al. [15] examined the relationship between perceived stress management skills, optimism and positive mood in 46 men who had undergone radical prostatectomy for localised PCA. While optimism was, not surprisingly, associated with positive mood, the relationship was mediated by the self-reported skills in stress management. The results support a focus on promoting strategies to deal with stress in any psychosocial intervention.

Few of the above investigations have enlisted large samples or controls, although we have noted three exceptions [5-7]. Nonetheless, we can state that PCA patients, examined at a single point, experience poor psychological adjustment in relation to specific symptoms, particularly sexual, bowel, pain and fatigue. They also exhibit psychological reactions not easily detected by broad measures. The question as to whether associations are a function of stage of disease, type of treatment or other factors remains unanswered. Data comparing distress between clinical subgroups are scanty [11].

### Longitudinal studies of psychological adjustment to PCA (see Table 2)

**Table 2:** Longitudinal studies of psychological adjustment to prostate cancer

**Study**	**Design**	**Characteristics of the sample**	**Major Findings**
Nordin et al. (2001)	• 6 months study with 2 observation points Measures include the Hospital Anxiety & Depression Scale and the Impact of Events Scale	• 522 cancer patients (mixed diagnoses, 118 with PCA, 8% late-stage PCA)	• Depression scores at diagnosis predicted levels 6 months later Risk factors: Advanced disease; low social support • Recommendation: Screen with a depression measure and one social support question
Visser et al. (2003)	• 3 months study with 2 observation points Measures include the EORTC	• 23 PCA and 38 benign prostate hyperplasia patients • Large attrition rates • Mixed therapy modes	• QOL for PCA patients decreased over the 3 months but not for BPH patients • Psychosocial factors were stable and contributed little to change in QOL

The demands of a longitudinal design have presumably militated against investigators tackling psychological adjustment over time as such studies are rare. We have identified a mere two, compared to the nine aforementioned cross-sectional studies. Nordin et al. [16] investigated depression and anxiety over six months following diagnosis. Levels of depression and anxiety at time of diagnosis were lower in the PCA than other cancer groups but were strong predictors of levels six months later, that is, those initially depressed or anxious remained so at follow up. Less social support outside the family and advanced disease at baseline also predicted depression and anxiety.

Visser et al. [17] examined QOL for three months following a PCA diagnosis and compared them with those of men diagnosed with benign prostatic hyperplasia (BPH). Only the cancer patients experienced reduced QOL. Importantly, the effect resulted from changed physical functioning; psychological factors contributed only marginally.

Longitudinal designs allow researchers to track the course of psychological adjustment but only one unreplicated finding emerges from the scanty above work – levels of depression and anxiety at the time of diagnosis identify those likely to be feeling depressed or anxious at follow-up. Screening and corresponding early intervention may therefore reduce longer-term psychological ill-effects but this ideal remains to be tested.

### Patterns of coping (see Table 3)

**Table 3:** Coping in men with prostate cancer

**Study**	**Design**	**Characteristics of the sample**	**Major Findings**
Bjork et al. (1999)	• Cross-sectional study – mail-out survey using the Mental Adjustment to Cancer Scale, The Life Orientation Test, Beck Depression Inventory and the Stait-Trait Anxiety Inventory	• 30 usable responses (mean 62 years since diagnosis) from 55 questionnaires distributed	• Completed a range of distress-related and self-esteem measures • Helplessness related to lower self-esteem; Loss appraisals to depression; threat appraisals to anxiety; optimism negatively related to depression and anxiety
Helgeson and Lepore (1997)	• Cross-sectional study – mail-out using the CARES and several measures developed for the study	• 162 usable responses from 258 questionnaires distributed Most (83%) were radical prostatectomies, a mean of 13 months since diagnosis	• Self-focused identity associated with worse functioning, greater cancer difficulties and poorer emotional expression • Expressed emotion mediated the links between self-identity and cancer difficulties
Lepore and Helgeson (1998)	• Cross-sectional study – mail-out using MHI-5, Impact of Events Scale, and CARES	• 181 usable responses from 258 questionnaires distributed Most (83%) were radical prostatectomies, a mean of 13 months since diagnosis	• Social constraints in talking about cancer moderated trauma and mental health relationships • Conclusion: Supportive social networks may promote psychological adjustment by facilitating cognitive processing of the cancer experience
Penedo et al. (2003)	• Cross-sectional study using the Life Orientation Test-Revised and Measure of Current Status	• 46 radical prostatectomy patients recruited to a stress management study	• Optimism, perceived stress management skills, and positive mood were correlated • Relationship between optimism and positive mood might be mediated by perceived stress management skills.
Zakowski et al. (2003)	• Cross-sectional study comparing men and women with cancer using the Social Constraints Scale, The Emotional Expressivity Scale, the Impact of Events Scale and the Profile of Mood States	• 41 men with PCA and 41 women with gynaecological cancer	• Men experienced greater distress in association with social constraints from their partners than did the women • Men might have fewer outlets for emotional expression so constraints from partners might lead to greater distress.

We found a relatively small number of 'acceptable' papers on coping, which is surprising given how relevant this topic is for psychological adjustment. Roesch et al., (2005), in their meta-analysis, included a greater number of studies, some of which did not meet our inclusion criteria. Their conclusions were that active, as opposed to more avoidant, approaches to coping with PCS are most beneficial for adjustment. Our review of this topic supports these conclusions.

Helgeson and Lepore [18] investigated the role that self-concept played in physical and emotional functioning. Men who focused on themselves, compared to those more interpersonally involved, had difficulty expressing emotions, which in turn could have mediated adjustment to their cancer.

The same group [19] examined men with PCA, diagnosed an average 17 months earlier. Those who were psychologically distressed found it difficult to talk about their condition with anyone. The authors speculated that support networks might reduce distress by allowing men to "process" their experience (a model familiar to those working in the trauma area). While these results are of interest, only prospective studies can illuminate the subject further.

Zakowski et al. [20] have provided an intriguing analysis of sex differences in emotional expression in cancer patients. While the results are limited by sample size [41 women with breast cancer and 41 men with PCA) and their inferences speculative, the findings warrant consideration. Expected differences between the men and women in terms of self-reported emotional expression as a means of coping emerged but these were not significant. However, the men reported that their spouses/partners were likely to be their main source of support whereas women had additional sources. Consequently, if men reported constraints on emotional expression they were likely to feel more distressed. Presumably, this was because women had more opportunities to express themselves, a strategy promoting adjustment. The possibility, that encouraging men's communication with their spouses, and beyond, could improve adjustment, is a promising one.

A common feature of coping research is to apply a standardised coping measure. A popular choice is the Mental Adjustment to Cancer (MAC) scale [21]. Bjork et al. [22] have used this, together with other measures, to investigate adjustment of men with PCA. With a small sample, and wide variation in the time between diagnosis and participation, validity is in doubt; results, however, are worth recounting. Helplessness was related to lowered self-esteem, appraisal of loss to depression, appraisal of threat to anxiety, and optimism, inversely, to depression and anxiety.

Results from these, and from other studies we excluded because of methodological limitations, have been integrated in a comprehensive meta-analysis [4]. Approach-type (problem-focused and emotion-focused) coping are associated with more positive psychological adjustment as well as better physical health in men with PCA. By contrast, avoidance-type strategies are linked to poor psychological adjustment and physical health. The evidence supports encouraging approach-type coping in any potential intervention.

Despite drawing this implication, potential research limitations must be borne in mind. Radically different ways of conceptualising coping have been adopted, using retrospective, cross-sectional designs. Researchers need to adopt a theory-based, prospective approach to study the relationship between coping and adjustment if the conclusions by Roesch et al. are to be confirmed.

### Treatments for PCA and psychological adjustment

Consensus has not been achieved as to indications of treatments in PCA. Moreover, decision-making is influenced by patients' attitudes to side-effects. As a corollary, adjustment assumes greater relevance. If a treatment is associated with side-effects such as fatigue and depression, overall psychological adjustment may deteriorate. Research on links between treatment and adjustment is clearly relevant. What follows is an account of this work, starting with surgery.

### Radical prostatectomy (RP) and psychological adjustment (see Table 4)

**Table 4:** Radical prostatectomy (RP) and psychological adjustment

**Study**	**Design**	**Characteristics of the sample**	**Major findings**
Pietroff et al. (2001)	• Retrospective, questionnaire – based inquiry, comparing those with (25%) and without PSA recurrence, an average 3 years post-surgery • UCLA Prostate Cancer Index and the RAND 36-item Health Survey used	• 348 patients with localized disease	• Small difference only on QOL between the 2 groups; generally, men well adjusted • PSA-recurrence group, unexpectedly had higher scores on emotional well-being
Rosetti and Terrone (1996)	• Retrospective inquiry (EORTC) 1 – 15 years after RP	• 161 patients, of whom "over 80%" did not have metastases	• Minimal overall psychological impact • 90% satisfied with surgery, and would opt to have it again
Heathcote et al. (1998)	• Retrospective inquiry 1–6 years after surgery • QOL measure constructed by authors	• 140 patients who had no evidence of recurrent or residual disease	• 90% satisfied with treatment, despite impotence in 40% of sample
Meyer et al. (2003)	• Retrospective inquiry (ED – QOL)a median 7 years after surgery	• 89 patients with localized disease	• Most patients felt adverse effects, including anger, guild and sadness associated with impotence. • 75% of men had lowered self-esteem to some degree
Ficarra et al. (2000)	• Retrospective inquiry an average 2 years after surgery; inclusion of control group of men who had RP for benign prostatic hyperplasia. • General Health Questionnaire and the Hospital Depression and Anxiety Scale	• 30 patients with localized disease	• Cancer patients had significantly higher levels of anxiety • Depression levels similar in 2 groups; minimal in each group
Randorf-Klym and Colling (2003)	• Retrospective inquiry 1–2 years following surgery • QOL measure constructed by authors	• 88 patients	• Perceived social support, self-esteem and health locus of control predicted post-surgery QOL • Suppression of anger and depression non-predictors

In the most impressive of seven studies found, involving 500 men [23], 70% completed questionnaires an average three years after surgery (no measures were taken at baseline). The mental health of those who did and did not experience recurrence was compared. Mental health was similar in the two groups, with overall satisfaction high. Thus, regardless of recurrence, men seem well adjusted, years after surgery.

In both an Italian survey of 161 men [21] and an Australian sample of 140 men [25], respondents mostly indicated minimal adverse effects in adjustment. Overall satisfaction was high. Sexual difficulties did not affect psychological functioning, suggesting that pursuit of cure outweighed sexual concerns.

British researchers [26], recognising that most erectile dysfunction questionnaires neglect psychological aspects, developed and validated a 15-item questionnaire, the Erectile Dysfunction Effect on QOL (ED-EQOL). They demonstrated retrospectively that surgery had a negative effect because of both direct physical factors and psychological influences. This study highlights the importance of using probing questionnaires to uncover subtle effects, rather than relying on diffuse survey tools.

Another Italian project [27] is less useful in that the sample was small. However, a control group undergoing surgery for benign prostatic hyperplasia was included. The PCA group had higher anxiety levels an average two years after surgery.

Rondorf-Klym and Colling [29] examined psychological adjustment 12 to 24 months after surgery. Perceived social support, self-esteem and locus of control all contributed to adjustment whereas age, sexual function and its appraisal, suppression of anger and depression did not.

The above studies suffer from two main limitations – follow-up ranging from one month to 15 years and psychological status, mostly assessed at a single point. Adjustment after surgery is tantamount to a journey – what patients encounter initially will differ from their experience at later points. Studies of adjustment over time are required, with distinctions made between immediate, intermediate and long-term stages.

### Radiotherapy (RT) and psychological adjustment (See Table 5)

**Table 5:** Radiotherapy (RT) and psychological adjustment

**Study**	**Design**	**Characteristics of the sample**	**Major findings**
Caffo et al. (1996)	• Retrospective "ad hoc" QOL questionnaire inquiry following RT	• 70 patients with localized PCA	• Psychological adjustment and relational well-being good • Level of available information about PCA and RT correlate with adjustment
Joly et al. (1998)	• Retrospective, controlled study of health-related QOL (EORTC)	• 71 patients with localized disease treated with combined external beam RT and brachytherapy	• Treatment has no adverse effects • Patients and controls similar on a range of measures but sexual and urinary symptoms more common in patients
Artebery et al. (1997)	• Retrospective questionnaire (EORTC Prostate) study following brachytherapy	• 51 patients with localized PCA	• Only a minority report psychological distress or disrupted social/family life • Return rate to work – 93% • 100% would recommend treatment to others
Monga et al. (1997)	• Prospective • Aim was to determine cause of fatigue in PCA patients receiving RT through questionnaire (Beck Depression Inventory and 2 sleep scales) and measure of neuromuscular efficiency (NME)	• 13 patients with localized PCA	• Significant but transient decline in NME, independent of psychological status; thus fatigue physically – based rather than influenced by depression
Monga et al. (1999)	• Prospective evaluation, including fatigue, at 4 points – before, during and after RT • Same measures as in Monga et al. (1997)	• 36 patients with localised PCA	• Fatigue scores significantly higher at end of treatment • Not associated with psychological status eg. depression or with sleep. • May be secondary to decline in neuromuscular efficiency and increased muscle fatigue
Greenberg et al. (1993)	• Prospective study of fatigue and mood (Beck Depression Inventory) during treatment	• 15 patients with localised PCA	• Fatigue increases with treatment, but independent of depression • Fatigue associated with increased interleukin – 1 which could be a marker for fatigue associated with RT

The five identified reports are uninformative. Considering the role of radiotherapy in PCA, this is surprising. An Italian study [30] is typical: 118 patients treated over a 13-year period were invited to participate. Of these, 28 refused, and 20 were ineligible. The outcome measure was *ad hoc*, comprising prostate-based items 'taken from different sources'. Follow-up ranged from months to years. Thus, assessment occurred in the initial stage of therapy or years later.

Given that brachytherapy (BT) is a recent development, its association with psychological functioning has barely been examined. French investigators [31] focused on the association between combined BT and external beam RT, and adjustment. In a rare design, controls were derived from electoral rolls. Men with localised PCA completed the questionnaires at one point, four to eight years after treatment. All scores were similar to those of controls, suggesting that patients are not adversely affected by RT/BT despite many experiencing loss of libido, decreased sexual activity, erectile difficulty and urinary incontinence.

Only one study has concentrated on BT as a single treatment modality [32]. In patients given a permanent transperineal radioactive implant, only a minority was psychologically impaired six months later. The pity is omission of a baseline measure, longer follow-up and controls. More work is needed on the psychological impact of BT, an increasingly common treatment for localised disease.

Two studies examined the association between RT and fatigue. As is common in psychiatric samples, fatigue could be due to depression. Monga et al. [33] examined this question in men with localised cancer using a repeated measures design. Fatigue did increase during RT but this was not associated with depression changes. A link however was found between fatigue and physical well-being. In another investigation [34], the team sought to determine the cause of fatigue and linked it to diminished neuromuscular efficiency, not psychological factors.

These studies of a prominent clinical feature like fatigue are worthwhile, particularly the effort to tease out links with psychological factors; useful psychological therapeutic implications are likely to emerge.

### Hormone therapy (HT) and psychological adjustment (see Table 6)

**Table 6:** Hormone therapy (HT) and psychological adjustment

**Study**	**Design**	**Characteristics of the sample**	**Major Findings**
Herr et al. (2000)	• Comparison of men treated with HT and men who defer such HT over 12 months • EORTC – Prostate	• 144 patients with locally advanced PCA or PSA – relapse after surgery or radiotherapy	• Men on HT had significantly more fatigue, anergia and emotional distress then men who deferred HT
Herr et al. (1993)	• Men choosing or postponing HT followed up for 6 months • EORTC – Prostate	• 35 patients with metastatic PCA	• Men on HT had more fatigue, psychological distress and sexual difficulties than those not on HT
da Silva et al. (1996)	• QOL (constructed by authors) examined in men on HT followed up for 12 months, rated by both patients and their physicians	• 63 patients with newly diagnosed PCA	• Poor correlation between the 2 sets of ratings • Fatigue, and emotional and social functioning, did not improve with HT
Green (2002)	• Study of QOL in PCA patient and control subjects over 6 months • Measures included EORTC and COPE Coping Scale	• 65 patients with non-localised PCA randomized to one of 3 forms of HT • 16 controls	• Emotional distress, self-efficacy and coping at baseline similar in treated and control groups • HT patients worse *or *better in various domains of QOL suggesting complex links • Overall, groups did not differ over time in psychological functioning
Stone et al. (2000)	• Sample followed up for 3 months after HT with focus on fatigue • Measures included EORTC, Hospital Depression and Anxiety Scale and 2 fatigue scales	• 58 patients, convenience sample, most with early stage disease	• Majority had significantly increased fatigue but not due to psychological factors (as was the case at baseline); rather due to diminished muscle function
Pirl et al. (2002)	• Men receiving HT for an average 3.3 years surveyed for depression • Measures included the Beck Depression and Fatigue Severity Scales	• 45 patients of whom 12 had metastatic disease.	• Major depression in 13%, 8 times the national rate in men • Past history of depression a risk factor for depressive reaction

Suppressing androgens is an accepted intervention in metastatic PCA but its role in less advanced disease is clouded given the complex issues involved – the goal of extending survival, occurrence of side-effects and impact on the psychological state.

The most noteworthy investigation of six identified is Herr and O'Sullivan's [35] follow-up of men with locally advanced PCA. While not a RCT, the men were offered orchidectomy, pharmacological androgen suppression or observation. Well-selected measures were administered at baseline, and at six and 12 months. Men receiving HT experienced significantly more distress than did those under observation. The finding raises the question of how to balance HT's adverse psychological effects with the potential benefit of extended life.

Herr et al. [36] also compared QOL in HT-treated and untreated men but on a sample diagnosed with metastatic PCA. Again, men were offered HT or observation and followed up for six months. The untreated group had better psychological adjustment than the HT-treated.

A similar study involved patients treated with orchidectomy or combined HT [37]. Measures covering fatigue and emotional and social functioning were completed at several points over 12 months. Although pain and urological symptoms improved, emotional and social functioning did not. This study stands out methodologically by virtue of obtaining both self-report and physician-based ratings. However, concordance was surprisingly poor.

In another study on men with metastatic PCA [38], but with the advantage of a comparison group, patients were randomised to one of three forms of HT or observation and completed psychological measures at baseline and six months later. As in Herr et al. [36] adjustment was similar across all groups, despite impaired sexual functioning in the hormone conditions. As part of their work, Green et al. [39] obtained patients' verbal comments and found substantial individual differences on such themes as health beliefs and coping. A detailed analysis revealed complex responses. Adjustment was worse in those with physical comorbidity, suggesting the relevance of health status in ascertaining adjustment associated with different treatments. HT enhanced *or *decreased adjustment. The position seems more intricate than conventional analyses reveal, and raises the question of whether broad brush-stroke patterns of psychological responses may mislead.

Another approach is to focus on a single psychological aspect. Stone et al. [9] considered fatigue. After three months of HT, fatigue had increased markedly but this was not related to adjustment but to diminished muscle function. Pirl et al. [40] selected depression in a heterogenous PCA group and found its prevalence eight times the national male rate. This was not associated with disease progression or use of chemotherapy but to a prior history of depression. Thus, history of disordered mood is a possible risk factor for depressive reactions in this group.

### Comparing different treatments and psychological adjustment (see Table 7)

**Table 7:** Comparing different cancer treatments and psychological adjustment

**Study**	**Design**	**Characteristics of the sample**	**Major findings**
Lee et al. (2001)	• Prospective 12 months assessment of QOL (Functional Assessment of cancer therapy – Prostate) after surgery, radiotherapy or brachytherapy	• Patients with localized disease: 23 treated with surgery, 23 with radiotherapy and 44 with brachytherapy	• After one month, virtually no change in emotional well being in all 3 groups • After 12 months, emotional well being similar to baseline in all 3 groups
Fossa et al. (2001)	• Prospective assessment of QOL (EORTC) at 6 weekly intervals until death	• 101 men treated with steroids, 100 with hormone therapy – all showing hormone-resistant metastatic PCA	• Men on steroids have better role functioning and less fatigue, especially between weeks 3 and 12. • Because of attrition thereafter, comparisons between the two groups not possible
da Silva (1993)	• QOL (EORTC – Prostate) assessed at 6 months by urologists and patients	• 76 men with metastatic PCA – treated with orchidectomy or hormone therapy	• Because of feasibility problems, comparative analysis not possible • Ratings by urologists correlate poorly with those of patients
Eton et al. (2001)	• Cross-sectional assessment of QOL (UCLA Prostate Cancer Index) within 7 weeks of launch of treatment.	• Men with localised PCA; 156 treated surgically, 49 with radiotherapy, and 51 with brachytherapy	• 3 treatment groups similar in psychological aspects • Support, self-efficacy and self-esteem predict better QOL
Litwin et al.	• Observational study of PCA (Cancer Rehabilitation Evaluation System and Functional Assessment of Cancer Therapy – General) patients and age and ZIP-code matched controls	• 214 localized PCA patients • 273 controls	• No differences in general QOL, including emotional well being, between surgery, radiotherapy and observation only sub-groups, or between PCA patients and controls
Fossa et al. (1997)	• Cross-sectional, retrospective assessment of QOL (EORTC)	• 379 men with PCA of various stages: 57 observed only, 112 received hormonal therapy, 96 surgery	• Sexual impairment and fatigue common in 3 treated groups but this does not have much effect on ratings of QOL
Lilleby et al. (1999)	• Controlled, cross-sectional assessment of QOL (EORTC) one year after treatment	• 154 men with PCA of various stages received radiotherapy, 108 surgery; 38 control	• Emotional function similar in 3 groups • Emotional function good or only slightly impaired in most patients
Cassileth et al. (1992)	• Prospective assessment of QOL (Functional Living Index-Cancer) and mood at 3 and 6 months follow-up	• 159 men with advanced PCA; 115 chose hormone therapy, 32 orchidectomy	• Mood improved at 3 months in both groups • Improvement greater in hormone therapy than orchidectomy patients at 6 months
Bokhour et al. (2001)	• Participation in focus group (7 groups); qualitative approach to QOL concerns • Men treated 12–24 months previously	• 48 men with early PCA treated with surgery, radiotherapy or brachytherapy	• Most men had sexual difficulties in terms of sexual relationships, intimacy and sense of masculinity • QOL – sexual effects – treatment type associations not mentioned
Van Andel et al. (2003)	• QOL (EORTC) assessed pre-treatment only	• 65 patients with localized disease treated surgically, 73 with radiotherapy	• Cognitive, but not emotional, function better in patients about to be treated surgically. Also especially in terms of sexual functioning and fatigue
Steginga et al. (2004)	• Prospective study before one of three treatments and two and 12 months after treatment • A range of psychological distress scales eg. Impact of Events Scale and Satisfaction with Life Scale	• 111 patients with localised disease – 56% treated surgically, 19% with RT and 25% with watchful waiting	• No differences found by medical treatment group in psychological adjustment at baseline or at follow-up. • Overall QOL similar to community norms

A typical study is by Lee et al. [41], in which patients with localised PCA were treated with surgery, RT or BT. They completed psychological measures before treatment and at several points during the next year. Poor adjustment was notable in the first month in the surgery and BT groups but not the RT group. At 12 months the difference had evaporated, with adjustment reverting to pre-therapy levels. The authors posited that patients should be counselled about differential treatment effects. We are less convinced, given that these were remarkably similar after three months. The non-randomised design, a serious limitation, precludes setting guidelines.

Few teams have adopted a prospective design. In a RCT comparing steroids and HT in men with metastatic PCA, Fossa et al. [42] examined psychological effects over six months. Patients on steroids were better off than their HT counterparts. However, patients dropped out in both groups because of deterioration in health and that the psychological difference was observed particularly from the third to the 12^th ^week. We may also note the authors' corollary that steroids should be considered as a first choice in hormone-resistant PCA but a brief comparison period because of attrition suggests a more tentative conclusion.

The prospective study by da Silva [43] compared adjustment after orchidectomy or HT over 12 months. However, attrition proved such a problem that comparisons were not feasible.

Steginga et al. [44] assessed psychological function before treatment – either RT, RP or watchful waiting – at two and 12 months later in men with localized PCA. The three groups showed improved adjustment over time but were similar at all three points. The relevance of obtaining baseline data is shown by the findings that QOL was similar to that found in the community.

Other studies in this area are cross-sectional, retrospective and lack baseline measures. The value of any findings is thus diminished. Eton et al. [45], for instance, compared adjustment to (non-randomised) RT, external beam RT and BT in men with localised PCA. Given the limited design, the three groups, not surprisingly, were similar in role function, perception of health and emotional well-being. A similar picture was found by Litwin et al. [46] in their comparison of RP, RT, observation alone and control.

A comparison of adjustment in watchful waiting, HT, RT and RP by Fossa et al. [47] (see their 2001 prospective study above) was also cross-sectional and retrospective. Despite physical morbidity in many patients, adjustment was rated as good. As the authors surmised, men with PCA seem to accept physical problems as the price for therapy that might cure.

Lilleby et al. [48] measured, cross-sectionally, adjustment of RT and RP a year following treatment. Desirable features were sizable samples and a control group (a watch and wait condition). Results were similar in both groups, with less than one in 10 reporting poor adjustment.

In another adequately sized sample, this time of advanced PCA patients, HT and orchidectomy were compared [49]. Adjustment improved after six months in the medical but not surgical group. Given that this was not a RCT and that follow-up was brief, proper comparisons are difficult, but results suggest surgical HT may be less appropriate than medical HT.

Two studies have examined the effects of treatments on sexual functioning. Bokhour et al. [50] formed focus groups of men who had undergone RT or BT for non-metastatic PCA sharing experiences of impotence. Although this method serves as a fertile source of qualitative material, pitfalls abound. Noteworthy here was sampling: men were recruited from medical centres but how they were selected is not stated, and only a third of 130 invitees participated. Nevertheless, the approach highlights the value of eliciting narratives, which may complement quantitative data. Mixed methods of data collection warrant serious consideration.

The importance of prospective studies incorporating baseline measures is vividly illustrated by Van Andel et al. [51]. They compared baseline profiles of men who received surgery or external beam RT. The latter reported worse functioning in physical, role, sexual and social domains. While these differences might have been attributable to treatment, it is critical to measure baseline psychological adjustment so that any pre-treatment discrepancies can be controlled.

### The effects of psychological interventions on adjustment (see Additional file 1)

Given a diagnosis of life-threatening cancer like PCA, the question arises as to whether interventions can be devised to help patients (and possibly partners) with such sequelae as existential concerns, poor coping and psychiatric morbidity. Considering the accomplishments in breast cancer [52], the dearth of studies in PCA is striking; only a few researchers have mounted an intervention to deal with psychological needs.

Educational programs are the most common but limited in what they set out to achieve. In an RCT, Helgesen et al. [53] compared "treatment as usual" with a program in which a trained nurse contacted the patient every six months over three years (the patient could also initiate contact) to explore general health and PCA-related difficulties. There were no differences at baseline or follow-up. The authors concluded that a nurse-based follow-up is cost-effective and "safe", i.e. nothing serious is missed. Little can be assumed about the psychological dimension, given the focus on nurse as "doctor surrogate".

Another nurse-based study concentrated on preparing patients for RT [54]. Routine nursing was offered as a controlled condition, consisting of a brief "tutorial" on the nature and side-effects of the treatment. The intervention was delivered at four points: pre-RT, during the first and last weeks of therapy, and one month following termination. Again, the goal was to inform the men about RT's nature and side-effects, but in much more detail, and the program also embraced psychological issues. The researchers only found less disruption of customary life activities in the intervention group compared with the controls.

Hack et al. [55] audiotaped the patient-oncologist consultation and compared its effects to a control group. Six weeks later, patients who received audiotapes were better able to recall information and were more satisfied with the level of communication. This is not an elaborate initiative but probably contributes to the doctor-patient alliance. Whether psychological well-being is affected remains unclear. A single tape might not exert much influence but, as part of a psycho-educational program, it might help as a means to empower the patient.

Another way to enhance doctor-patient communication is through printed information. Templeton and Coates [56] conducted an RCT in which men with PCA receiving hormone therapy were randomly assigned to receive an education package or to a control condition. Significant differences were found on knowledge about PCA and its treatment and on QOL but the groups used similar coping strategies. The absence of disease staging and only a one-month follow-up are notable limitations.

Lepore and colleagues [57] examined group-based education in men with localized PCA, comparing its effects with a controlled condition and a more ambitious intervention – an educational group plus discussion. The interventions were held weekly for six weeks. At one-year follow-up, the "education plus discussion" patients were less bothered by sexual difficulties and more likely to be in steady employment than the other patients. The authors' speculation that the opportunity to discuss personal problems confers extra benefits is intuitively appealing.

Mishel et al. [58] examined the moderating effects of variables such as race, education and number of sources of information on two psycho-educational interventions for localised PCA patients. Those with less education benefited by increasing their knowledge; men with more sources of information also did better. These results support implementing interventions that tailor content and teaching methods to specific needs.

Only two teams [59,60] have tested more substantial interventions, based on cognitive behaviour therapy (CBT) strategies. In the former RCT [59], less than half the patients were satisfied or reported any benefit after an average 3.6 sessions. The study is replete with limitations: only one in four invitees participated; data are presented for treated patients but not for controls; treatment ranged from one to 24 sessions, despite a set limit of eight; no baseline assessments were obtained; and the sample was heterogeneous (localised and metastatic disease).

The RCT by Petersson et al. [60] was substantially better in design and execution. Treatment over eight weekly sessions (and a booster) contained education, CBT and relaxation. Psychological distress three months after commencement improved compared to controls but this was confined to copers who actively sought information in contrast to those who avoided doing so, identifying a moderator of benefits. A snag is the range of therapeutic components; the question remains as to which lead to change.

Much has been written about support groups (SGs) in cancer, particularly of the breast, but PCA does not feature particularly. We have found only four papers, and none of them illuminating. The differential gender attention could relate to the possibility that men are less inclined to share their health concerns with others.

Krizek et al. [61] asked men with PCA about their experience of, and attitudes to, SGs. Only one in eight had attended one or more meetings, but those who had, did so an average 10 times, and valued sharing concerns. In-depth enquiry of reasons for not attending might have proved informative.

It would be worthwhile repeating such a study with a representative sample and a more comprehensive form of enquiry. Poole et al. [62] have accomplished this to a degree. They administered questionnaires to PCA patients recruited though SGs and radiotherapy/oncology clinics. Not unexpectedly, both SG members and non-participants identified a spouse/partner as most helpful in providing emotional and practical support. The former relied on SG members for information, the latter on doctors. Possibly because of the prominent role of spouse/partner, no differences emerged between the two groups regarding coping and adjustment. SGs seem limited in their effects, although they do need to be evaluated in a RCT.

Gregoire et al. [63] did not apply such a design in their study of men (as well as "some" relatives) recruited to participate in 10 weekly sessions led by a nurse and a psychologist, which concentrated on information about PCA, its effects and its treatments. Men who attended regularly rated the experience highly since it helped them to cope more effectively. The authors pointed to evidence for the utility of SGs despite conceding the dubious representativeness of the sample and lack of baseline data.

Only one study has examined the value of an actual SG intervention [64]. The researchers hypothesised that men with PCA, facing the prospect of RT who received information about the treatment, would cope more effectively than controls. Taped material was given to men at four points, each containing accounts by former patients of their RT experience. One outcome variable differentiated intervention from control groups – the former were far less disrupted in their customary recreational activities during and after treatment. The groups, however, were similar in terms of negative mood, this probably attributable to normal baseline levels. A similar study with more emotionally distressed patients is clearly indicated.

Johnson [65] has extended this work by looking at the role of optimism-pessimism. In a similar RCT, information exerted a positive effect in pessimistic patients. Again, intervention led to less disruption of recreational activities, this time in both optimistic and pessimistic men.

An interesting variation of the SG emerges from the pilot work of Weber et al. [66] who recruited 10 long-term (three plus years) PCA "survivors" of RP to serve as partners over eight weekly sessions to men who had undergone surgery for PCA. The latter were randomised to this intervention or to a control condition. Self-efficacy increased in the peer-supported men but not in the controls but benefit in terms of depression was limited. Although the small sample precluded adequate statistical treatment the self-help rationale is sound and may suit men who are hesitant about participating in a group.

In arguing the need for an RCT to test the efficacy of SGs, we are not suggesting neglect of other methods. Qualitative research, for instance, enables us to learn about group therapeutic factors involved. A good example is Arrington's [67] participant observation. Interested in how the men dealt with sexuality, he observed a disproportionate emphasis on performance but was cognisant of three limitations: excluding the perspective of spouses/partners, thereby neglecting relational dynamics; using a restricted sample of men from a retirement community; and the fluctuating nature of group membership. Notwithstanding, the approach complements traditional methods and further qualitatively-based research is worthwhile.

The quality of research on psychological interventions varies. Considerably more sophisticated therapies are needed through which psychological distress can be addressed. While education and problem solving have a place, RCTs directed to such features as depression, anxiety, marital strain and poor coping are needed.

## Discussion

Although this review reveals many gaps in knowledge, embryonic patterns are emerging. Cross-sectional studies comparing men with PCA and controls suggest few differences in levels of psychological distress. But we must appreciate that PCA patients constitute a heterogeneous group and that adjustment is better in those with localised disease compared to those with advanced forms.

Longitudinal studies do not suggest that adjustment worsens over time. However, early signs of distress, particularly depression and anxiety, indicate a poorer psychological prognosis. Coping that involves interpersonal awareness and expression of emotions is linked to better adjustment. By contrast, a sense of helplessness correlates with low self-esteem and depression.

### Implications for clinicians

Clinicians would be advantaged if able to detect distress in their patient and in the couple, as well as to identify those at risk, and in knowing about programs that alleviate psychological complications. Little knowledge has accumulated thus far. We can only rely on low-level evidence such as that emerging from observational studies.

Men with PCA do not appear to experience marked impairment of adjustment; the rate is much less than that of women with breast cancer. As is well established in men's health research, low rates of morbidity may reflect a belief that admitting to personal difficulties and the need for help is a "sign of weakness" [68]. Moreover, questionnaire-based designs may fail to penetrate these "avoidant" responses.

An alternative to the questionnaire is the focus group. Qualitative data may be as valuable as quantitative data. Thus, in the study by Bokhour [50], participants felt able to talk about sexual impotence and related concerns. The typical limitation is the poor generalization of findings.

Given the low rate of psychological morbidity, the clinician's ability to identify predictors would at least enable them to monitor "at risk patients". At this stage, however, statements about predictors cannot be made. Longitudinal studies with adequate methodology are essential to address this question and these are rare.

We had anticipated differential effects on adjustment of the medical treatments offered to men with PCA but outcomes tend to be uniform. We should remember, however, that they tend to start with low levels of distress. A corollary is the role of psychological outcomes in decision-making about treatment. If it were the case, for instance, that a specific treatment affected mood adversely, clinicians could point this out in the consent process.

One study alerts us to the dangers of possible artefacts in calculating mean scores for presumed homogeneous populations. Green [38] found a mixed pattern when examining the psychological effects of hormone therapy – one subgroup improved, another deteriorated. Moderating factors are elusive but their likely relevance makes it imperative to identify them.

Even if we could identify those at risk, the literature does not guide the clinician to interventions of proven value. This aspect is the most scanty of all those researched. Educational strategies and support groups have negligible influence. The role of psychotherapy is unclear, since trials are rare, and limited methodologically. For the moment, the clinician must resort to ideographic appraisal regarding the sort of help a patient requires.

### Implications for researchers

The implications that follow mirror the limitations noted throughout the review, namely: sampling, design, controls, outcome measures and statistical methods.

The most striking feature is the need for studies of adequate standard. Consider sampling: larger, more representative and well-described samples are required, e.g., age, socio-economic status, ethnicity, education, stage of cancer and treatment received. A key strategy is multi-site recruitment. Even then, a sample with enough power takes time to generate; selection criteria guarantee that.

Aspects of design also need attention. Cross-sectional "snapshots" are of limited value in that they fail to tell us how long symptoms persist and who is vulnerable to enduring maladjustment. A snapshot at the point of diagnosis is bound to be influenced by reactions on learning one has a life-threatening illness. Similarly, assessment following one or another treatment will be affected by the nature of that treatment. Only prospective, longitudinal, repeated measures designs can illuminate the patient's journey and identify risk factors.

Control groups are crucial even though they present difficulties, especially for an observational study. In any treatment study, a control condition is essential if results are to be taken seriously. Recruitment might be less thorny than for prevalence studies; the ethical dilemma of not providing treatment is not insurmountable since there is no current psychological standard intervention for men with PCA.

A close examination of psychometric issues is beyond the scope of this paper but valid and reliable measures to describe the psychological features of PCA patients and to use as outcome criteria are required. The variegated and, in some cases, home-baked measures encountered has precluded confident interpretation of the results emerging from their use. Researchers need to concentrate on well-established instruments, like the Beck Depression and the Brief Symptom Inventories, and evaluate them in a concerted way.

Qualitative work is a valuable complement to quantitative research. There is scope to encompass thematic analyses in future research, especially with the advent of sophisticated software to examine patients' narratives.

Statistical methods have improved substantially in recent years and need to be applied in future work. Consider two of many possible illustrations: sampling and differential outcome. In allocating men with PCA to different arms of a RCT, it will be vital to do so with rigor, including the need for stratification. We will need to examine a range of sub-groups such as the varied outcomes in different clinical groups of patients, e.g., on a spectrum of severity of psychological distress.

## Conclusion

Psychological adjustment to PCA has attracted growing interest of investigators but the yield over the past decade has, in contrast to the picture in breast cancer, been meagre, both in terms of the number of studies carried out and their methodological rigour. If future research is to prove more useful, the bar will need to be raised. Fortunately, several leads have emerged; the time has arrived to make the most of them.

## Supplementary Material

Additional File 1 Table 8: Effects of psychological interventions on adjustment Click here for file
