# Supplementary material for: Psychological adjustment of men with prostate cancer: a review of the literature
Source: Biopsychosoc Med. 2007 Jan 10;1:2. doi: 10.1186/1751-0759-1-2 (PMC1805773; doi:10.1186/1751-0759-1-2)
Supplement: Additional File 1 — Table 8: Effects of psychological interventions on adjustment [file 1751-0759-1-2-S1.doc]

# Table 8: Effects of psychological interventions on adjustment

| **Study** | **Design** | **Characteristics of the sample** | **Major findings** |
| --- | --- | --- | --- |
| Helgesen et al.  (2002) | - Patients randomised to follow-up by specialist nurse or usual practice, ie urologist - 3 year follow-up - Assessment included Hospital and Anxiety Depression Scale | - PCA in any stage, 200 in nurse group and 200 in comparison group | - Frequency of complications similar in the 2 groups - Lag time from symptoms to intervention similar in the 2 groups - Levels of anxiety and depression similar in the 2 groups - Both approaches equally cost-effective |
| Johnson et al.  (1997) | - Quasi-experimental with repeated measures including Life Orientation Test and Profile of Mood States - Experimental group received regulation theory – based interventions on 4 occasions from staff nurses – tutorials on nature and side-effects of radiotherapy as well as topics like expectations - Control group received standard nursing care | - 226 patients receiving radiotherapy for breast cancer or PCA (stage not clear) | - Experimental condition patients experienced less disruption in usual life activities during and following radiotherapy compared to controls |
| Hack et al.  (1998) | - Patients randomly assigned to standard care or to audio-taped consultation with oncologist, the tape then given to the patient - Measures included State Anxiety Inventory | - 36 patients with breast cancer (18) or PCA (18), in any stage | - Recall of information significantly greater in group receiving audiotape - Those with most recall of information at follow-up had highest level of satisfaction with patient-doctor communication |
| Lepore et al.  (2003) | - Patients randomized to receive 6 weekly sessions of education or education plus discussion or to control condition | - 93 patients with localized PCA in each condition | - Education plus discussion condition superior in terms of sexual difficulties and job record |
| Hellbom et al.  (1998) | - Patients randomised to individual psychological support (IPS) group in which cognitive – behavioural methods applied or to a control group - Satisfaction with IPS measure | - 527 patients newly diagnosed with breast, colorectal, gastric and PCA (stage unclear) | - Average 3.6 sessions of treatment. Less than half of PCA patients were satisfied or reported benefit at termination |
| Krizek et al.  (1999) | - Telephone interview of patients with breast cancer or PCA about their attitudes to support groups | - 87 patients with PCA, 130 with breast cancer (stage not clear) | - Men less likely than the women with breast cancer to join a support group (13% v 33%) - Men who do join attend for about a year – similar to women |
| Poole et al.  (2001) | - Questionnaire study of patients with PCA regarding support and coping | - 234 patients with PCA(stage not clear) – 142 attend support groups, 92 did not | - Attendees see other patients as sources of emotional, informational and practical support compared to non-attendees - No significant differences between attendees and non-attendees regarding coping and satisfaction with 3 types of support |
| Gregoire et al.  (1997) | - Participants in support groups (10 weekly sessions) asked to rate satisfaction with experience - Program focused on psychological reactions (Profile of Mood States) to, and coping, (Sickness Impact Profile) with the cancer | - 54 men with PCA (all stages) and “some family members” | - Participants felt they understood illness better and were more involved in their treatment, but no baseline data collected |
| Johnson et al.  (1989) | - Randomized controlled trial of support group in which nature of radiotherapy discussed versus comparison group, during a course of radiotherapy | - 84 men with localized PCA undergoing radiotherapy; 42 randomized to intervention, 42 to control condition | - Intervention patients far less disrupted in their usual activities, but groups similar in mood |
| Weber et al.  (2004) | - Pilot study - Patients randomized to control condition or to a program in which they met on 8 weekly occasion with long term PCA survivor (> 3 years) - Measures included Stanford Inventory of Cancer Patient Adjustment and Geriatric Depression Scale | - Patients who underwent radical prostatectomy (details of cancer not cited) | - Program proved feasible - Self-efficacy increased in supported men but effects on depression and social support limited |
| Johnson  (1996) | - Randomized controlled trial - Patients allocated to: - intervention-focus either on self-care and coping or on concrete objective information or to control condition | - 62 PCA patients receiving radiotherapy for localized cancer | - Intervention associated with less disruption in usual activities, in both pessimistic and optimistic patients - Concrete information intervention had positive effect on mood in pessimistic patients |
| Arrington  (2000) | - Participant observation of support groups over 3 years - Special interest on how participants deal with sexuality - Application of grounded theory to qualitative data | - Patients with PCA attending support group (stage not clear) | - Restricted perspective on sexuality, with disproportionate emphasis on performance - Group leader, a physician, could have steered the men away from sharing other aspects of sexuality. When psychologist lead the group, sexuality theme tackled more readily |
| Petersson et al.  (2002) | - Randomised controlled trial - Treatment group met for 8 weekly sessions (but mixed with gastrointestinal cancer patients) - 2 education, 6 cognitive-behavioural, also relaxation - Outcome measured 3 months after launch of treatment-Impact of Event Scale, Hospital Anxiety and Depression Scale | - 59 patients received treatment, 59 controls - stage not cited | - Treated patients improved on measures compared to controls but only in those who were identified at baseline as information-seeking rather than information-avoidant |
| Mishel et al.  (2003) | - Effectiveness study of potential moderators of 2 psycho-educational interventions - Patients randomly assigned to one of 2 treatment groups or 2 control groups - Potential moderators measured at baseline | - 239 men with PCA; 44% were Afro-American | - Men with less education benefited by increasing their knowledge of PCA - More sources of PCA-related information was related to better outcome - Lower level of religiosity was related to better participation in treatment planning |
| Templeton and Coates  (2004) | - Patients randomised to educational package or usual practice - 1 month follow-up using the Functional Assessment of Cancer Therapy-Prostate Instrument and Jalowiec Coping Scale | - 28 in experimental group, 27 controls, all receiving hormonal therapy - Staging of PCA not obtained | - Experimental condition men achieved better knowledge of disease and its treatment, and superior QOL compared to controls - No difference on use of coping strategies |
